# Chemical effect of silver (Ag) and yttrium (Y) co-doping on silicon-based fullerene (Ag@Si_59_Y) sensor nanostructures: a computational adsorption study of cyanogenic halide gases†

**DOI:** 10.1039/d5ra03374h

**Published:** 2025-07-28

**Authors:** Idongesit J. Mbonu, Gideon E. Mathias, Emily O. Udowa, Zainab Abbas Abd Alhassan, Thamer A. A. M. Alalwani

**Affiliations:** a Department of Chemistry, Federal University of Petroleum Resources Effurun Nigeria; b Department of Pure and Chemistry, University of Calabar Calabar Nigeria mathiasgideon610@gmail.com; c Mazaya University College Dhiqar Iraq; d Radiological Techniques Department, College of Health and Medical Techniques, Al-Mustaqbal University 51001 Babylon Iraq; e Biomedical and Computational Chemistry Group, University of Calabar Calabar Nigeria

## Abstract

Cyanogenic gases, such as hydrogen cyanide and cyanogen, are highly toxic and pose serious risks to both human health and the environment. Effective adsorption strategies are essential to mitigate these hazards. In this study, the adsorption potential of a silver-decorated and yttrium-doped silicon nanocluster (Ag@Si_59_Y) toward cyanogenic gases—BrCN, ClCN, and FCN—was investigated using density functional theory (DFT) at the ωB97XD/GenECP/LanL2DZ/Def2SVP level of theory. Adsorption was explored in two orientations for each gas molecule. The computed adsorption energies indicated favorable interaction, particularly for BrCN, with values of −30.121, −17.571, −17.571, −16.943, −16.316, and −16.316 kcal mol^−1^ for BrCN–Br–, BrCN–N–, ClCN–Cl–, ClCN–N–, FCN–F–, and FCN–N–Ag@Si_59_Y complexes, respectively. BrCN showed the strongest affinity, suggesting preferential adsorption on the Ag@Si_59_Y surface. Noncovalent interaction (NCI) analysis and recovery time calculations confirmed the presence of strong chemisorptive interactions, especially for BrCN, characterized by significant charge transfer and bonding stability. The frontier molecular orbital (FMO) analysis revealed a notable reduction in the energy gap upon gas adsorption, highlighting the enhanced reactivity of the surface. High dipole moment values across all adsorbed complexes indicate substantial charge separation, which is advantageous for sensor-based applications. Furthermore, the Electron Localization Function (ELF) analysis provided visual insight into the nature of bonding interactions. ELF maps exhibited moderate to high localization around the adsorption regions, particularly in BrCN–Br–Ag@Si_59_Y and FCN–N–Ag@Si_59_Y, suggesting mixed covalent and noncovalent bonding characteristics. These observations corroborate findings from QTAIM and NBO analyses, validating the interaction types and reinforcing the reliability of the proposed adsorption mechanisms. Thus, Ag@Si_59_Y demonstrates strong and selective adsorption properties toward cyanogenic gases, making it a promising candidate for use in gas sensing and environmental detoxification technologies.

## Abstract 2

Cyanogenic gases, such as hydrogen cyanide and cyanogen, are highly toxic and pose serious risks to both human health and the environment.

## Graphical abstract

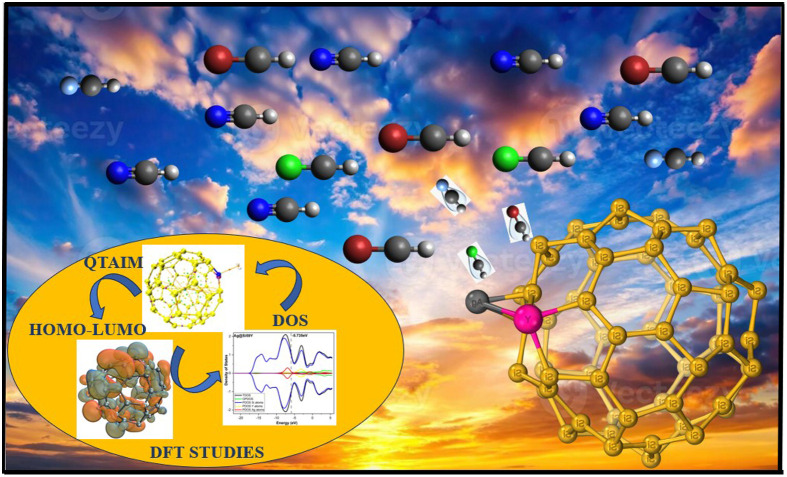

## 1 Introduction

Cyanide exposure poses severe dangers, leading to rapid respiratory failure, cardiovascular collapse, and, at high doses, fatal poisoning. By disrupting cellular respiration, it presents immediate and life-threatening risks to individuals.^1^ Primarily, cyanide gas refers to gaseous substances containing cyano groups, such as hydrogen cyanide (HCN), cyanogen chloride (CNCl) and dicyan ((CN)2).^2^ Research has shown that little volatile and poisonous cyanide gas is present in the atmosphere and is created as a result of industrial and human activities.^3^ Its primary sources are the use and processing of compounds containing cyanide, the burning of fossil fuels derived from coal, the pyrolysis of biomass, the burning of materials used in building décor, and the denitrification of exhaust gas.^4^ Cyanide gas is commonly available and reasonably priced as a precursor used in chemical processes; hence, it is not impossible that it will be used in future conflicts and terrorist attacks. Commonly found in cyanide gas, HCN and CNCl have been employed in military settings as systemic toxic agents.^2,4^ On the basis of its increasing danger to individual health and well-being, there is a growing need to build effective adsorbents for cyanide gas.^5^ For example, the adsorption of cyanides onto appropriate materials, such as activated carbon, is often employed in processes such as wastewater treatment to remove these harmful compounds.^6^ Absorption methods also involve long processing cycles, often limiting their practical application. Therefore, more efficient environmentally friendly methods for cyanide gas elimination are needed in the future.^2^

Interestingly, the application of computational techniques, notably density functional theory (DFT), has revolutionized the assessment of nanomaterials as adsorbents.^7^ DFT enables precise predictions of adsorption properties, aiding in the identification of highly effective nanomaterials for diverse applications.^8^ This approach accelerates the design process, optimizing the selection of nanomaterials with enhanced adsorption capacities and facilitating advancements in environmental remediation, gas sensing, and storage devices.^8^ Chen *et al.* employed DFT calculations to evaluate the potential of nitrogen- and oxygen-comodified nanotubes for the adsorption of nitrogen oxide and reported that O–N CNTs could maintain a stable structural morphology during the adsorption of gas molecules.^9^ The adsorption of polluting gas molecules (NO_2_, SO_2_, and O_3_) on the surface of Al-doped stanene nanotubes was examined by Karimi *et al. via* density functional theory (DFT) calculations. The findings demonstrated that NO_2_, SO_2_, and O_3_ were adsorbed on the Al-doped stanene nanotube and that the Al-doped stanene nanotube was able to detect all three gas molecules with different detection strengths.^10^ Density function theory (DFT) was used by Jasim *et al.* to determine the geometrical and adsorption properties in their study, and the authors reported that the optimal adsorption energy in the investigation was 0 eV because all the interaction strengths produced positive values.^11^ In light of this, it is clear that computational analyses yield valuable insights for constructing potent adsorbent materials. DFT stands out as a reliable predictor, enhancing our ability to design and develop effective adsorbents.^8^ Recognized for its accuracy, DFT plays a pivotal role in advancing the creation of materials with better adsorption capabilities, fostering breakthroughs in environmental remediation and gas sensing.^7^

Additionally, the systems' adsorption capacities are enhanced by the alteration of the nanomaterials. This, therefore, makes them appropriate for possible applications.^12^ Solimannejad *et al.*, explores the adsorption of cyanogen gas on Si_12_C_12_ and Cu-decorated Si_12_C_12_ fullerenes using DFT calculations.^13^ The results show weak physisorption on pristine Si_12_C_12_ but Cu decoration enables chemisorption with significant charge transfer and improved sensitivity for detection of cyanogen gas. Moses Abraham in 2021 investigated the adsorption of cyanogen halides (X–CN; X = F, Cl, Br) on pristine and Fe/Mn-doped C_60_ using DFT study. The results shows weak physisorption on pristine surface while strong chemisorption on doped surfaces was observed, revealing reduced band gaps and enhanced sensitivity.^14^ Another study by Castrucci *et al.,* synthesized silicon nanotubes (SiNTs) using a gas phase condensation technique, confirming their existence through various characterization methods such as TEM and SEM.^15^ The findings reveal unique structural properties and hybridization characteristics, indicating that silicon fullerene, can be experimentally synthesized in the laboratory. In the present research, silver (Ag)-decorated and yttrium (Y)-doped silicon fullerene were evaluated *via* density functional theory for their geometric, electronic, topological, and adsorption properties for the adsorption of cyanogenic gases (Br–CN, Cl–CN, and F–CN). Both cyanides and certain halides can be harmful gases, and their adsorption is a significant consideration in various contexts, particularly in industrial processes and environmental protection.^16–19^ However, the adsorption of harmful gases such as cyanides and certain halides is a practical strategy in various applications to minimize their impact on human health and the environment.^20–22^ Prior investigations have revealed that group IV atom-doped gold clusters (MAu_16_^−^, M = Si, Ge, Sn) exhibit exohedral structures stabilized by strong dopant–gold interactions, suggesting that such doped systems can serve as highly reactive sites for adsorption processes. We emphasize that this research is poised to achieve promising outcomes, revealing potential adsorbents capable of mitigating the impact of cyanide gas.^23^ This study will effectively address questions regarding efficacy, offering valuable insights into the development of strategies to minimize the harmful effects associated with cyanide exposure.

## 2 Computational methodology

In this study, the GaussView 6.0.16 and Gaussian 16 software were used for modelling and geometry optimization of the Ag@Si_59_Y nanocage surface using the ωB97XD functional along with the GenECP basis set combination. This basis set includes LanL2DZ for heavy atoms (Ag, Y) and Def2SVP for lighter atoms (C, F, Cl, H).^24^ The ωB97XD functional incorporates long-range dispersion corrections, making it suitable for accurately modelling intermolecular interactions.^25^ To determine the most stable (ground-state) structure, several initial configurations were generated by systematically varying the positions of the Ag and Y dopants on the Si_59_ nanocage. Each configuration underwent full geometry optimization at the aforementioned level of theory. The structure with the lowest total energy and no imaginary frequencies was selected as the ground-state geometry. Although full global optimization methods such as genetic algorithms or basin-hopping were not implemented due to computational limitations, our local optimization approach sufficiently sampled diverse initial geometries to ensure structural stability and energetic favorability.

Electronic properties, including conductive behavior and stability, were analyzed using frontier molecular orbital (FMO) theory, and stabilization energies were obtained from natural bond orbital (NBO) analysis *via* NBO 7.0 implemented in Gaussian 16.^26^ HOMO and LUMO iso-surfaces were visualized using Chemcraft software, while the density of states (DOS) plots were computed using Multiwfn and visualized *via* origin.^27–29^ Furthermore, non-covalent interaction (NCI) plots were generated using VMD software to provide qualitative insight into the adsorption mechanism between the surface and cyanogenic gas molecules.^30,31^

The adsorption energy for cyanogenic gases on the nanocage surface was calculated *via*eqn (1).1
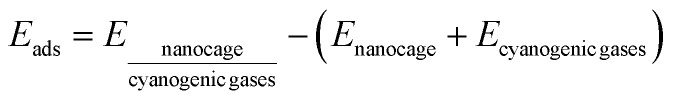
*E*_nanocage/cyanogenic gases_ represent the total energies of the cyanogenic gases adsorbed and the nanocage, and *E*_nanocage_ and *E*_cyanogenic gases_ represent the total energies of the adsorbed gases and isolated nanocage, respectively.

## 3 Results and discussion

### 3.1 Structure of gas molecules

**Fig. 1:**
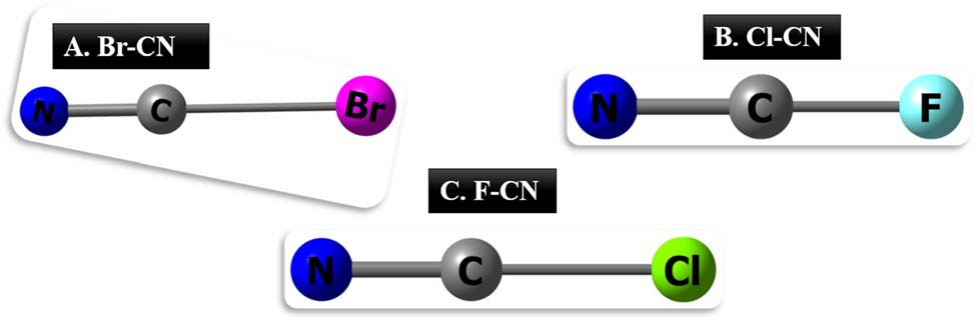
A pictorial display of the studied cyanogenic gases; A. (BrCN), B. (Cl-CN), C. (F-CN).

In this section, we discuss the geometric properties of cyanogenic gases. Notably, the cyanogenic gases (BrCN, ClCN, and FCN) were first optimized separately. Consistent with prior research, the C–N bond length is 1.47 Å, and the triple bond length is 1.16 Å. For Br–CN, the C–N bond decreases to 1.46 Å, whereas the Br–C bond is observed at 1.91 Å. Conversely, the Cl–CN bond between C–N slightly contracts to 1.15 Å, which is smaller than that observed for both Br–CN (C–N 1.46 Å) and F–CN (C–N 1.49 Å). Intriguingly, the F–C bond is notably shorter at 1.25 Å, whereas the Br–C bond appears longer at 1.91 Å, with the Cl–C bond observed at 1.63 Å. The variation in bond lengths can be attributed to the electronegativity of the atoms involved and their impact on bond strength. Fluorine (F) is highly electronegative, creating a stronger pull on the shared electrons in the F–C bond, resulting in a shorter bond length.^28^ In contrast, bromine (Br) is less electronegative, leading to a weaker attraction between the atoms and a longer Br–C bond.^29^ The interplay of electronegativity and atomic size influences the observed differences in bond lengths among these cyanogenic gases. See Fig. 1 for a graphical illustration of the cyanogenic gases.

### 3.2 Structure and stability of surface and gas molecules

**Fig. 2:**
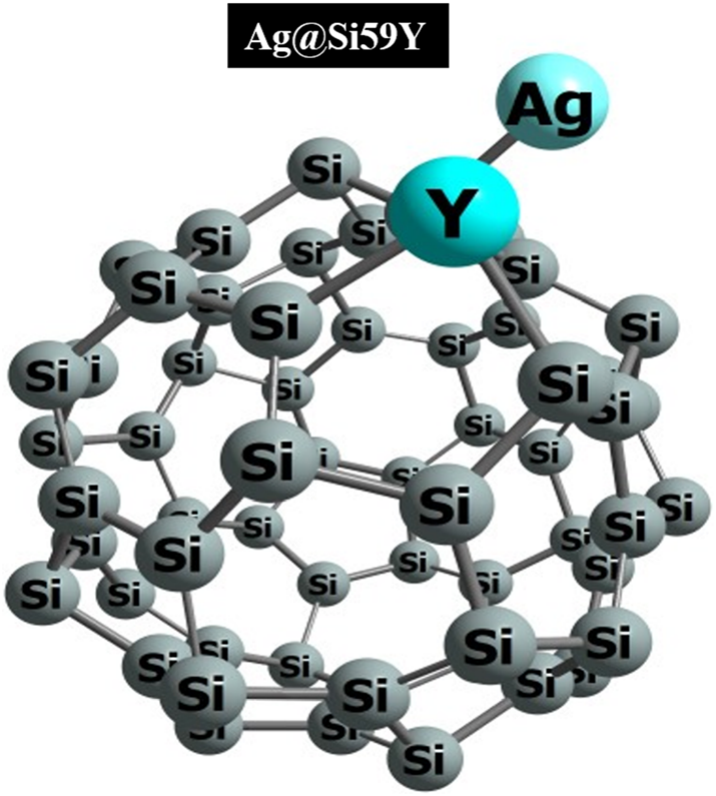
Optimized and stable structure of Ag@Si_59_Y.

First, nanocages are a molecular framework with hexagonal and pentagonal rings that form their structure. This unique architecture imparts exceptional stability and versatility, making nanocages promising for different applications.^32^ Initially, our examination focused on the structure and stability of Ag@Si_59_Y. Fig. 2 illustrates the stabilized configuration of the aforementioned surface. The Si nanocage has distorted bonds due to the effects of the metal-decorated atom (Ag) and the yttrium (Y) dopants. The Si–Si bonds in the hexagonal ring and pentagonal ring are 2.71 Å and 2.34 Å long. While the bond between the dopant and Y–Si is found at 2.71 Å, the bond between Ag–Si is reported at 2.53 Å.

**Fig. 3:**
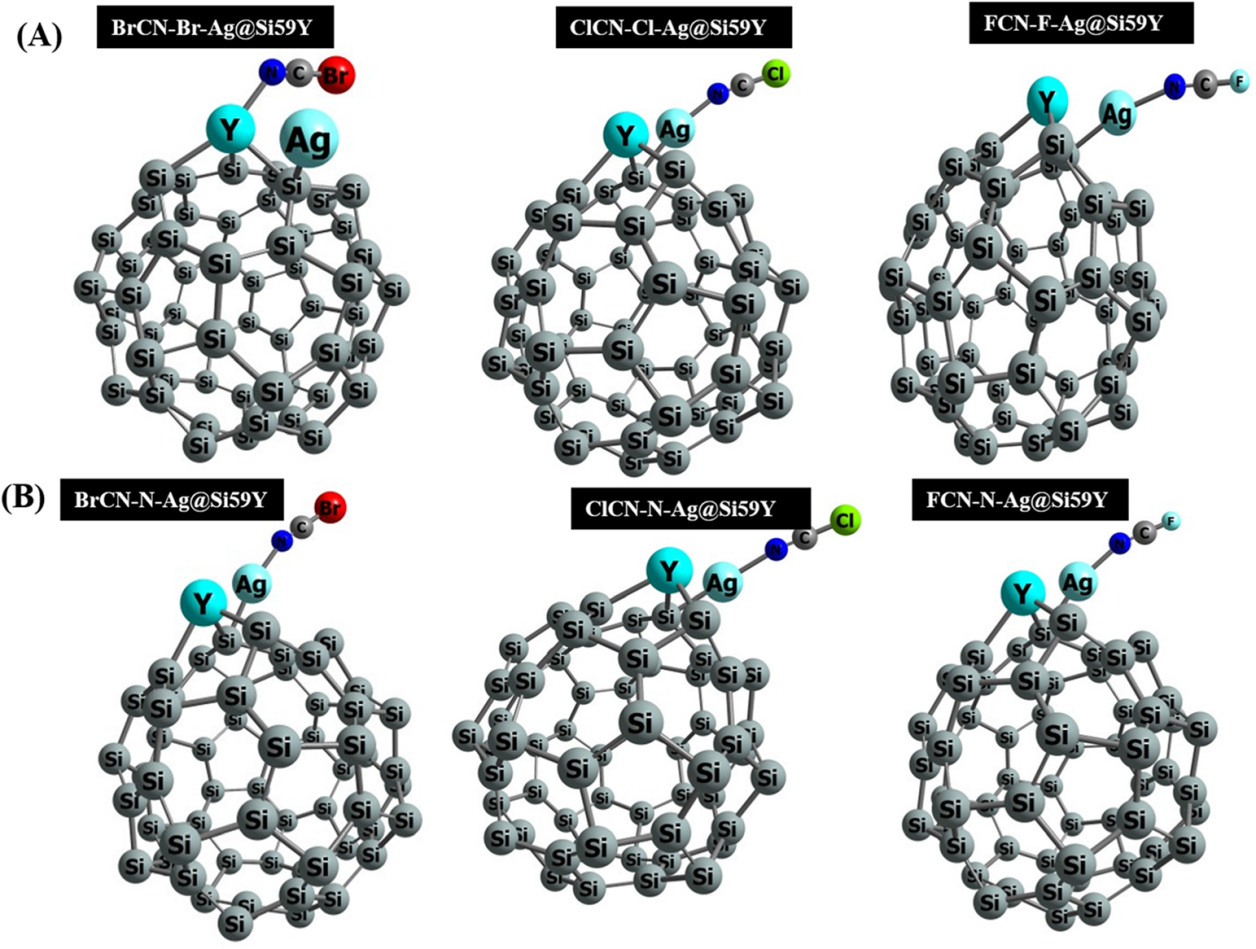
Pictorial view of relaxed and stable complexes; (A) (BrCN-Br-Ag@Si59Y, ClCN-Cl-Ag@Si59Y, FCN-F-Ag@Si59Y) (B) (BrCN-N-Ag@Si59Y, ClCN-N-Ag@Si59Y, FCN-N-Ag@Si59Y).

Thereafter, the gas molecules were oriented in two (2) positions on the adsorbing surface. One configuration positioned the surface adjacent to the Br, Cl, and F atoms from the gas molecule on the right, whereas the other positioned the N atom on the left side of the adsorbing surface (see Fig. 3).^33^ Notably, the Si–Si bond lengths in all the complexes were revealed. This crucial structural characteristic is consistently found between 2.20 Å and 2.60 Å. At the same time, 2.53 Å is also found for the Ag–Si bond length. This consistency points to a strong adsorption pattern, emphasizing the complexes' stability and coherence in their interactions with cyanogenic gases.^34^ The Si–Si and Ag–Si bond lengths are important markers of the structural stability and efficiency of the adsorption process inside these complexes.

### 3.3 Vibrational analysis

Table 1 shows the vibrational frequencies for BrCN, ClCN, and FCN, both before and after adsorption on Ag@Si_59_Y. The reported frequencies reflect experimental values from the literature,^35,36^ whereas the observed frequencies are derived from theoretical calculations. Before adsorption on Ag@Si_59_Y, the observed C–Br stretching frequency of 602.57 cm^−1^ falls within the reported frequency range of 700–600 cm^−1^. After adsorption, the peak frequency increased to 653.79 cm^−1^, indicating effective interaction with the surface. The slight reduction in frequency compared with the reported range may suggest a weakening in the C–Br bond due to electron donation from the silver surface or steric effects from the surrounding silicon framework. The C=N stretching frequencies before and after adsorption (2338.56 & 2336.00 cm^−1^) exceeded the reported range (2280–2200 cm^−1^), indicating significant interactions between BrCN and Ag@Si_59_Y. This shift may be attributed to charge transfer, thereby strengthening the C=N bond upon adsorption. The Cl–C stretching frequency before and after adsorption (753.77 & 606 cm^−1^) of the ClCN molecule falls within a close range with the reported frequency, suggesting effective adsorption. The slight downshift indicates that ClCN experiences similar electronic effects as BrCN after adsorption on Ag@Si_59_Y. The C=N stretching frequency (2356.42 cm^−1^) is higher, indicating a strong interaction with the surface and a stabilization effect from Ag@Si_59_Y. The observed frequency of F–C stretching for the FCN molecule (1152.95 cm^−1^) is significantly lower than expected. The frequencies associated with Si–Si stretching ranged from 592.46 to 606.63 cm^−1^, suggesting minimal disruption to the silicon framework after adsorption of the cyanogenic gases. This stability is crucial for maintaining sensor performance and indicates the potential of Ag@Si_59_Y to adsorb gases without significant structural distortions. Following adsorption, a redshift in C=N, as indicated by higher frequencies for all the gases, suggests that these molecules will reach stability on the Ag@Si_59_Y surface. The observed shifts in the C–Br and C–Cl stretching frequencies indicate that these interactions are influenced by both steric and electronic factors.

**Table 1:** Comparison of observed and experimentally reported vibrational frequencies for BrCN, ClCN, and FCN adsorption on the Ag@Si_59_Y nanostructured surface

Interaction	Assignment	Reported frequency	Observed frequency
Br–C–N	C–Br	700–600	602.57
	C=N	2280–2200	2338.56
Cl–C–N	Cl–C	800–700	753.77
	C=N	2280–2200	2356.42
F–C–N	F–C	2317–1060	1152.95
	C=N	2280–2200	2439.10
Ag@Si_59_Y-freq	Si–Si	620–600	606.04
BrCN–Br–Ag@Si_59_Y	C=N	2280–2200	2336.00
	C–Br	700–600	653.79–601.48
	Si–Si	620–600	592.46
BrCN–N–Ag@Si_59_Y	Si–Si stretching	620–600	603.09–606.53
	C–Br	700–600	647.08
	N=C	2280–2200	2373.40
ClCN–Cl–Ag@Si_59_Y	Si–Si stretching	620–600	603.09
	Cl–C	800–700	804.52
	C=N	2280–2200	2394.91
ClCN–N–Ag@Si_59_Y	Si–Si stretching	620–600	606.63
	Cl–C	800–700	804.65
	C=N	2280–2200	2392.74
FCN–F–Ag@Si_59_Y	Si–Si stretching	620–600	602.96
	F–C	900–1300	1186.22
	C=N	2280–2200	2523.60
FCN–N–Ag@Si_59_Y	Si–Si	620–600	571.14
	F–C	900–1300	1186.17
	C=N	2280–2200	2523.61

### 3.4 HOMO–LUMO analysis

In line with previous research, the frontier molecular orbital energies, which encompass the highest occupied molecular orbital (HOMO) and lowest unoccupied molecular orbital (HOMO), which are responsible for accepting and donating electrons, are also essential in determining how a molecule interacts.^7,27^ Notably, this characteristic can be elucidated from the difference between the two orbital energies, which is often referred to as the energy gap (*E*_g_) expressed in electron volts (eV). Additionally, the stable and conductive properties of the system and complexes can be explained by the values obtained from the Eg.^7,8^ The results for the HOMO, LUMO, and *E*_g_ of the system and complexes are expressed in Table 2. First, there is uniformity in the HOMO and LUMO values observed for all the systems. However, pristine Ag@Si_59_Y has an Eg of 4.20 eV; for BrCN complexes, BrCN–Br–Ag@Si_59_Y has an *E*_g_ of 4.05 eV, whereas BrCN–N–Ag@Si_59_Y has an *E*_g_ of 4.02 eV. Thus, with a greater energy gap for Ag@Si_59_Y, the surface is more stable than the BrCN complexes are, whereas the lower energy gap observed for BrCN–N–Ag@Si_59_Y makes it more conductive than BrCN–Br–Ag@Si_59_Y is. Similarly, the ClCN–Cl–Ag@Si_59_Y complex has a similar energy gap of 4.02 eV as that found for BrCN–N–Ag@Si_59_Y, which implies that both surfaces exhibit similar adsorptive characteristics. Additionally, ClCN–Cl–Ag@Si_59_Y is more conductive than ClCN–N–Ag@Si_59_Y because of its lower energy gap of 4.03 eV. Seemingly, the FCN complexes are observed with the same energy gap of 4.02 eV for both FCN–F–Ag@Si_59_Y and FCN–N–Ag@Si_59_Y. Our results revealed that a similar energy gap was observed for the complexes, suggesting that their adsorptive characteristics are consistent with each other. The reduction in the energy gap compared with that of the pristine system shows that the system is sensitive to cyanogenic gases.

**Table 2:** Calculated HOMO, LUMO, energy gap and quantum descriptors for the studied system and complexes computed *via* the DFT/ωB97XD/GenECP/LanL2DZ/Def2svp computational method

Systems	HOMO eV	LUMO eV	*E*_g_ eV	*η* (eV)	*S* (eV^−1^)	*μ* (eV)	*χ* (eV)	*ω* (eV)
Ag@Si59Y	−6.71	−2.68	4.20	2.10	0.24	−4.70	4.70	5.24
BrCN–Br–Ag@Si_59_Y	−6.63	−2.58	4.05	2.03	0.49	−4.60	4.60	5.23
BrCN–N–Ag@Si_59_Y	−6.52	−2.50	4.02	1.34	0.25	−4.51	4.51	5.05
ClCN–Cl–Ag@Si_59_Y	−6.53	−2.51	4.02	1.34	0.25	−4.52	4.52	5.08
ClCN–N–Ag@Si_59_Y	−6.49	−2.46	4.03	2.01	0.50	−4.48	4.48	4.98
FCN–F–Ag@Si_59_Y	−6.55	−2.53	4.02	2.01	0.248	−4.54	4.54	5.12
FCN–N–Ag@Si_59_Y	−6.55	−2.53	4.02	2.01	0.248	−4.54	4.54	5.12

**Fig. 4:**
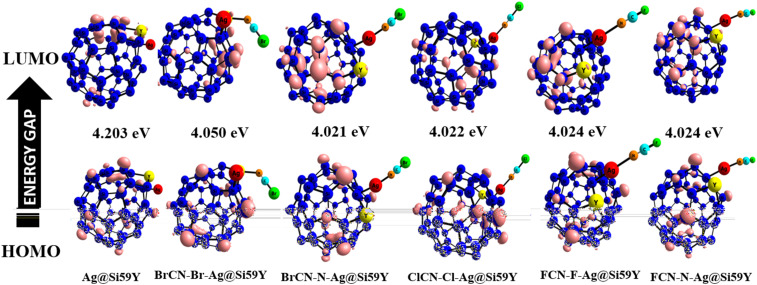
HOMO and LUMO surface plots for the studied system and complexes.

To gain further understanding of the electronic characteristics of the system and its complexes, quantum descriptors such as chemical hardness (*η*), chemical softness (*S*), chemical potential (*μ*), electronegativity (*χ*), and electrophilicity (*ω*) derived from the HOMO and LUMO values offer nuanced insights.^29^ The calculated descriptors not only reveal a remarkable consistency in the HOMO, LUMO, and energy gap values across all entities but also signify their comparable adsorptive behaviors (see Table 1). This uniformity in descriptors suggests coherent electronic behavior within the system and its complexes, showcasing their analogous interactions with quantum states. The obtained quantum descriptors serve as a strong framework for deciphering and comparing the electronic intricacies, paving the way for a comprehensive understanding of the adsorption phenomena within this intricate molecular framework.^12,13^ The isosurface plots of the highest occupied molecular orbital (HOMO) and lowest unoccupied molecular orbital (LUMO) are presented in Fig. 4, which clearly depict that the orbital distributions correspond flawlessly with the obtained results. This consistency supports the validity of the results that are acquired and illustrates well-balanced electronic behavior in the system and its complexity.^14^ This offers a thorough and visually pleasing depiction that supports the consistency found in the quantum descriptors and electrical properties among the studied entities.

The density of states (DOS) analysis (ESI, Fig. S1†) reveals consistent electronic behavior across the studied complexes, confirming stable adsorption characteristics of the cyanogenic gases. The observed DOS profiles exhibit distinct energy level distributions, with variations attributable to the influence of halide substituents (F, Br, Cl) on the electronic structures. These findings align with frontier molecular orbital (FMO) results and further substantiate the comparable adsorptive behavior among the complexes.

### 3.5 NBO analysis

Natural Bond Orbital (NBO) analysis is a robust quantum chemical technique employed to investigate intra- and intermolecular interactions, hyperconjugative effects, and stabilization mechanisms within molecular systems.^37^ It provides a clear understanding of bonding characteristics by analyzing electron density distributions and evaluating second-order perturbation stabilization energies (*E*^2^), which reflect the strength of donor–acceptor interactions between filled (donor) and vacant (acceptor) orbitals.^31,32^ These interactions are closely linked to electronic properties such as dipole moments and polarizability, offering insights into reactivity and adsorption behavior.^27–30^ In this study, the second-order perturbation energies (see Table 3) show dominant π → LP* and π → π* transitions across the systems. For the pristine Ag@Si_59_Y surface, the strongest interactions were observed in transitions such as πSi_3_–Si_4_ → LP(1)Si_2_ (51.66 kcal mol^−1^), πSi_35_–Si_39_ → LP(1) Si_40_ (47.94 kcal mol^−1^), and πSi_14_–Si_56_ → LP(2)*Si_23_ (41.21 kcal mol^−1^), which reflect the surface's inherent electronic delocalization and adsorption potential. Upon adsorption, BrCN–N–Ag@Si_59_Y displayed the highest stabilization energies among all complexes, with values such as 79.90, 51.76, and 40.85 kcal mol^−1^, corresponding to π → LP* and π → π* transitions (*e.g.*, πSi_37_–Si_38_ → π*Si_37_–Si_38_ and πSi_21_–Si_24_ → LP(3)Si_20_). These enhanced donor–acceptor interactions correlate directly with the most negative adsorption energy (−30.121 kcal mol^−1^), indicating strong chemisorption and higher selectivity for BrCN.

**Table 3:** Most contributing stabilization energies and their donor and acceptor orbital transitions for the studied system and complexes

System	Donor (*i*)	Acceptor (*j*)	*E* ^2^ kcal mol^−1^	*E*(*i*)−*E*(*j*)	*F*(*i*, *j*)
Ag@Si_59_Y	πSi_3_–Si_4_	LP*Si_2_	51.66	0.14	0.084
	πSi_35_–Si_39_	LP*Si_40_	47.94	0.16	0.083
	πSi_1_–Si_6_	LP*Si_2_	47.88	0.17	0.086
	πSi_14_–Si_56_	LP*Si_23_	46.39	0.14	0.079
	LP*Ag_61_	LP*Ag_51_	41.21	0.12	0.109
BrCN–Br–Ag@Si_59_Y	πSi_37_–πSi_38_	LP*Si_36_	61.16	0.16	0.096
	πSi_21_–πSi_24_	LP*Si_20_	51.76	0.16	0.096
	πSi_45_–πSi_46_	π*Si_28_–πSi_41_	40.85	0.19	0.080
	πSi_9_–πSi_10_	LP*Si_36_	38.94	0.16	0.077
	πSi_1_–πSi_2_	π*Si_50_–πSi_51_	31.61	0.22	0.074
BrCN–N–Ag@Si_59_Y	σSi_57_–Ag_61_	LP*Y_60_	79.90	0.36	0.155
	πSi_16_–Si_19_	LP*Si_20_	50.69	0.16	0.088
	πSi_21_–Si_24_	LP*Si_20_	48.92	0.16	0.084
	πSi_26_–Si_27_	πSi_15_–Si_22_	47.25	0.18	0.087
	σSi_57_–Ag_61_	σ*Si_57_–Y_60_	45.31	0.63	0.166
ClCN–Cl–Ag@Si_59_Y	πSi_16_–Si_19_	LP*Si_20_	50.76	0.16	0.088
	πSi_21_–Si_24_	LP*Si_20_	49.14	0.16	0.084
	πSi_11_–Si_12_	π*Si_8_–Si_13_	39.48	0.20	0.081
	LP Cl_64_	π*C_62_–N_63_	38.55	0.56	0.132
	LP N_63_	LP*Ag_61_	29.02	0.73	0.131
ClCN–N–Ag@Si_59_Y	πSi_26_–Si_27_	π*Si_15_–Si_22_	47.19	0.18	0.087
	πSi_21_–Si_24_	LP*Si_20_	46.60	0.16	0.084
	LP*Y_60_	LP*Y_60_	42.06	0.03	0.078
	σ*C_62_–N_63_	π*C_62_–N_63_	41.95	0.11	0.185
	πSi_15_–Si_22_	π*Si_16_–Si_19_	41.38	0.19	0.081
FCN–F–Ag@Si_59_Y	π Si_16_–Si_19_	LP*Si_20_	51.00	0.16	0.088
	σSi_57_–Ag_61_	LP*Si_20_	49.58	0.20	0.095
	σSi_57_–Ag_61_	σ*Si_57_–Y_60_	46.74	0.63	0.169
	LPF_64_	π*C_62_–N_63_	42.65	0.73	0.158
	πSi_15_–Si_22_	π*Si_16_–Si_19_	42.14	0.19	0.081
FCN–N–Ag@Si_59_Y	πSi_21_–Si_24_	LP*Si_20_	49.42	0.16	0.084
	σSi_57_–Ag_61_	σ *Si_57_–Y_60_	46.73	0.63	0.169
	LPF_64_	π*C_62_–N_63_	42.60	0.73	0.158
	πSi_15_–Si_22_	π*Si_16_–Si_19_	42.14	0.19	0.081
	LPN_63_	LP*Ag_61_	30.55	0.74	0.135

In contrast, ClCN–N–Ag@Si_59_Y and FCN–N–Ag@Si_59_Y exhibited relatively lower stabilization energies and less negative adsorption energies (−16.943 and −16.316 kcal mol^−1^, respectively). This trend reflects weaker electronic coupling and donor–acceptor interaction between these gases and the surface, corresponding to reduced adsorption strength and selectivity. These findings highlight a clear correlation between the nature and magnitude of donor–acceptor interactions and the observed adsorption behavior. Specifically, systems exhibiting stronger π → LP* or π → π* transitions with higher stabilization energies tend to show more favorable adsorption energetics. Thus, the orbital interaction profile derived from NBO analysis provides mechanistic insight into the adsorption selectivity of the Ag@Si_59_Y surface toward different cyanogenic gases.

### 3.6 Topology analysis: QTAIM

To elucidate the nature of intermolecular interactions critical to the adsorption process, the Quantum Theory of Atoms in Molecules (QTAIM), as developed by Bader, offers a robust framework for characterizing bond types and strengths.^38^ By examining parameters such as electron density *ρ*(*r*), its Laplacian ∇^2^*ρ*(*r*), kinetic energy density *G*(*r*), potential energy density *V*(*r*), total energy density *H*(*r*), and bond ellipticity (*ε*), QTAIM helps distinguish between covalent, noncovalent, and partially covalent interactions.^28,34,35^ In this study, bond critical points (BCPs) were identified for various atomic interactions between the adsorbates (cyanogenic gases) and the modified Ag@Si_59_Y surfaces. According to QTAIM criteria, a covalent bond is characterized by ∇^2^*ρ*(*r*) < 0 and *H*(*r*) < 0, a noncovalent interaction by ∇^2^*ρ*(*r*) > 0 and *H*(*r*) > 0, and a partially covalent interaction by ∇^2^*ρ*(*r*) > 0 and *H*(*r*) < 0. The *G*(*r*)/|*V*(*r*)| ratio further refines this categorization: covalent (*G*(*r*)/|*V*(*r*)| < 0.5), partially covalent (0.5–1.0), and noncovalent (*G*(*r*)/|*V*(*r*)| > 1.0).^11^ Our results, summarized in Table 4, confirm that most bonds between the adsorbates and the Ag@Si_59_Y surface exhibit covalent characteristics, with *G*(*r*)/|*V*(*r*)| < 0.5 and *ε* < 1.0, indicating stable bonding configurations. For example, in the BrCN–Br–Ag@Si_59_Y complex, the Br_64_–Si_23_ bond exhibits a low ellipticity (*ε* = 0.151), along with ∇^2^*ρ*(*r*) = −0.709 and *H*(*r*) = −0.330, clearly indicating a covalent and stable bond. Similarly, for BrCN–N–Ag@Si_59_Y, the Y_60_–Si_67_ (*ε* = 0.205) and Si_4_–Si_57_ (*ε* = 0.149) bonds also display ∇^2^*ρ*(*r*) < 0 and *H*(*r*) < 0, reinforcing their covalent nature. In the ClCN–Cl–Ag@Si_59_Y system, bonds Si_4_–Si_9_ (*ε* = 0.167) and Si10–Si_11_ (*ε* = 0.173) also conform to covalent characteristics.

**Table 4:** Selected QTAIM topology analysis for the investigated complexes

System	Bond	*ρ*(*r*)	∇^2^*ρ*(*r*)	*G*(*r*)	*V*(*r*)	*H*(*r*)	*G*(*r*)/|*V*(*r*)|	*ε*
BrCN–Br–Ag@Si_59_Y	Br_64_–Si_23_	0.705	−0.709	0.136	−0.467	−0.330	0.291	0.151
BrCN–N–Ag@Si_59_Y	Y_60_–Si_67_	0.722	−0.752	0.142	−0.509	−0.367	0.278	0.205
	Si_4_–Si_57_	0.698	−0.736	0.124	−0.448	−0.324	0.276	0.149
ClCN–Cl–Ag@Si_59_Y	Si_4_–Si_9_	0.698	−0.736	0.124	−0.448	−0.324	0.276	0.167
	Si_10_–Si_11_	0.688	−0.687	0.129	−0.447	−0.318	0.288	0.173
ClCN–N–Ag@Si_59_Y	Si_14_–Si_17_	0.804	0.167	0.397	−0.374	0.225	1.061	0.419
	Si_27_–Si_32_	0.779	0.161	0.382	−0.361	0.211	1.058	0.216
FCN–F–Ag@Si_59_Y	Si_18_–Si_54_	0.691	−0.701	0.127	−0.448	0.320	0.283	0.163
FCN–N–Ag@Si_59_Y	Si_4_–Si_5_	0.682	−0.648	0.134	−0.457	−0.323	0.293	0.200

**Fig. 5:**
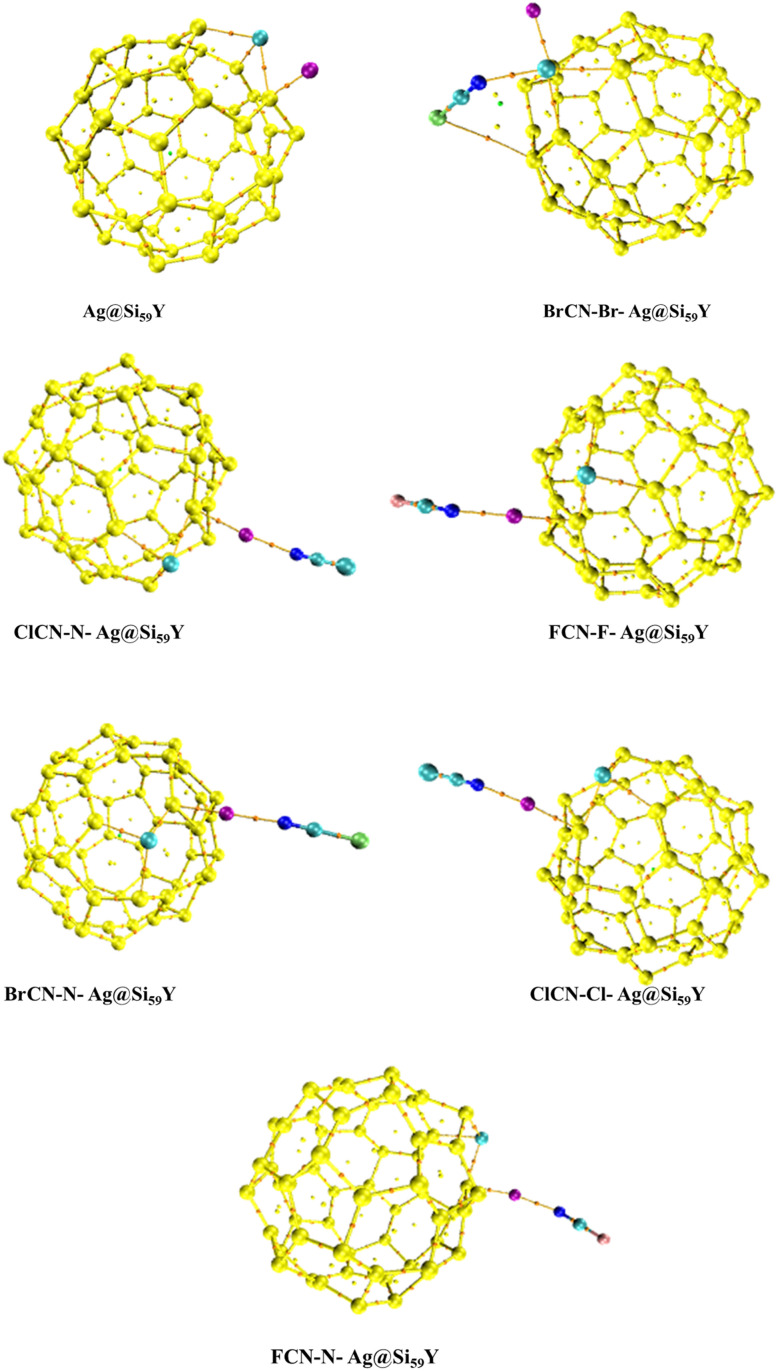
Graphical representation of QTAIM analysis showing intermolecular and intramolecular interactions at the bond critical points (BCPs) on the investigated surfaces.

However, in ClCN–N–Ag@Si_59_Y, the Si_14_–Si_17_ (*ε* = 0.419) and Si_27_–Si_32_ (*ε* = 0.216) bonds show ∇^2^*ρ*(*r*) > 0 and *G*(*r*)/|*V*(*r*)| > 1 (1.061 and 1.058, respectively), indicating noncovalent interactions, despite relatively low ellipticity values. These exceptions are attributed to the influence of chlorine's electronegativity and its steric interaction with the Si framework. For the FCN complexes, FCN–F–Ag@Si_59_Y shows a covalent Si_18_–Si_54_ bond (*ε* = 0.163), and FCN–N–Ag@Si_59_Y has a covalent Si_4_–Si_5_ bond (*ε* = 0.200), both with ∇^2^*ρ*(*r*) < 0 and *H*(*r*) < 0. Bond ellipticity (*ε*) values across all complexes remained below 1.0, indicating stability of the formed bonds and thus of the adsorbent systems. High ellipticity values (>1) would suggest bond instability or delocalization, but this is not observed in our results.^12^ The observed values underline the robust binding interaction between adsorbates and substrates. Overall, the QTAIM analysis provides strong evidence that the adsorption of cyanogenic gases onto the modified Ag@Si_59_Y surfaces is predominantly governed by covalent interactions, with specific atomic pairs playing key roles in stabilizing the complexes. The variation in bonding behavior, especially with Cl substitution, underscores the influence of halide type on adsorption mechanisms (Fig. 5).

### 3.7 Adsorption studies

**Fig. 6:**
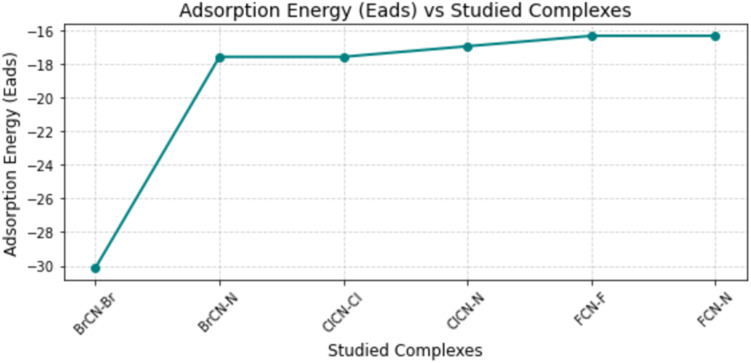
Adsorption energy (in kJ mol^−1^) of halogenated cyanides on the substrate, where more negative values suggest stronger interactions.

The energy of adsorption plays a crucial role in the development of sensing materials for gas detection. Calculating the adsorption energy involves determining the difference in energy levels between the nanocages and the nanocage-gas energies (see eqn (1)).^39^ This calculation provides valuable insights into the adsorption mechanisms and factors influencing the adsorption energy. Adsorption energies of −30.121, −17.571, −17.571, −16.943, −16.316 and −16.316 kcal mol^−1^ were computed for BrCN–Br–Ag@Si_59_Y, BrCN–N–Ag@Si_59_Y, ClCN–Cl–Ag@Si_59_Y, ClCN–N–Ag@Si_59_Y, FCN–F–Ag@Si_59_Y, and FCN–N–Ag@Si_59_Y adsorption, respectively (see Table 5). This reveals that the tendency of the nanocage to adsorb cyanogenic gases is greater for bromine cyanides than for chlorine and fluorine cyanogenic gases. This is explained by the chemically oriented adsorption energy observed between bromine cyanide and the Ag@Si_59_Y nanocage. Delving deeper into the adsorption sites of bromine cyanide, the interaction is stronger through the bromine atom than through the nitrogen atom, as explained by their adsorption energies of −30.121 kcal mol^−1^ and −17.571 kcal mol^−1^, respectively, for BrCN–Br–Ag@Si_59_Y and BrCN–N–Ag@Si_59_Y. The increased adsorption of bromocyanide compared with that of chlorocyanide and fluorocyanide can be attributed to several factors.^40^ First, the greater chemical affinity of bromine cyanide to the Ag@Si_59_Y nanocage surface may originate from its unique bonding characteristics, resulting in stronger interactions. Additionally, bromine is less electronegative than chlorine and fluorine are, which may contribute to more favorable interactions.^41^ The size and molecular structure of bromine cyanide may be more suitable for effective adsorption on the nanocage surface, thereby affecting the adsorption energy. Owing to the electronic structure of bromine, the effective charge transfer between bromine cyanide and Ag@Si_59_Y nanocages can further enhance the interaction. Furthermore, the electronic configuration of bromine and potential steric effects may together contribute to a more stable adsorption configuration. Similarly, ClCN exhibited moderate adsorption, with chlorine binding at −17.571 kcal mol^−1^ and nitrogen slightly weaker at −16.943 kcal mol^−1^. For FCN, both fluorine and nitrogen sites displayed the weakest adsorption energies of −16.316 kcal mol^−1^, likely due to fluorine's smaller size and lower polarizability. The overall trend shows that adsorption strength follows the order BrCN > ClCN > FCN, with halogen sites consistently more favorable than nitrogen sites. Essentially, a combination of chemical properties, electronegativity, molecular structure, charge transfer capabilities, electronic configuration, and steric effects may underlie the observed increased adsorption of bromine cyanide due to its interaction with the nanocage surface. Preferential interactions provide a comprehensive explanation. It is noteworthy that both BrCN–N–Ag@Si_59_Y and ClCN–Cl–Ag@Si_59_Y exhibit the same adsorption energy (−17.571 kcal mol^−1^). This value was carefully verified and confirmed not to be a typographical error. The similarity stems from a coincidental balance in interaction components (electrostatic, dispersion, and orbital contributions), despite differences in the interacting atoms and adsorbate nature. While numerically identical, QTAIM and NBO analyses reveal that the bonding environments and charge distributions differ across these systems. Also, the high adsorption energy recorded for BrCN on Br–Ag@Si_59_Y (−30.121 kcal mol^−1^) indicates strong chemisorption (see Fig. 6 and Table 5). While this suggests excellent selectivity and firm capture of BrCN, it may also pose challenges for desorption and sensor regeneration. In practical gas sensing applications, such strong binding might hinder rapid reversibility. However, this trade-off is sometimes desirable for detecting hazardous species at trace levels. Future work could explore thermal or photonic stimuli to facilitate desorption, or consider surface modification strategies to adjust the adsorption strength for enhanced reusability. The basis set superposition error (BSSE) was corrected using the counterpoise method to ensure accurate estimation of interaction energies between the gas molecules and the nanocage. All BSSE values are reported in Table 5, confirming their significant contribution to the corrected adsorption energies.

**Table 5:** Calculated adsorption energies for the studied complexes computed *via* the DFT/ωB97XD/GenECP/LanL2DZ/Def2svp computational method

	*E*_complex_/a.u.	Adsorbent/a.u.	Adsorbate/a.u.	*E*_ads_/eV	*E*_ads_/kcal mol^−1^	BSSE kcal mol^−1^
BrCN–Br–Ag@Si_59_Y	−19924.88	−17258.22	−2666.61	−1.31	−30.12	−27.23
BrCN–N–Ag@Si_59_Y	−19924.86	−17258.22	−2666.61	−0.76	−17.57	−14.95
ClCN–Cl–Ag@Si_59_Y	−17811.01	−17258.22	−552.76	−0.76	−17.57	−14.42
ClCN–N–Ag@Si_59_Y	−17811.01	−17258.22	−552.76	−0.74	−16.94	−14.12
FCN–F–Ag@Si_59_Y	−17450.67	−17258.22	−192.42	−0.70	−16.32	−13.19
FCN–N–Ag@Si_59_Y	−17450.67	−17258.22	−192.42	−0.70	−16.32	−13.19

### 3.8 Thermodynamic studies

The strength of the interactions between the cyanogenic gases (BrCN, ClCN, and FCN) on the surfaces of Ag@Si_59_Y can be further evaluated with vital thermodynamic parameters such as Gibbs free energy and enthalpy to distinguish the chemical behavior of the distinct systems by the principles of heat, work and physical changes.^37^ The spontaneity of the adsorption process can aid in determining the viability and potential application of surfaces for the adsorption of gases.^38^ The electronic energy (*ε*_0_), zero-point energy correction (*εZPE*), thermal correction to energy (*E*_tot_), thermal correction to enthalpy (*H*_corr_), thermal correction to free energy (*G*_corr_), *ε*_0_ + zero-point energy, *ε*_0_ + thermal energy correction, *ε*_*0*_ + thermal enthalpy correction, *ε*_0_ + thermal free energy correction, change in Gibbs free energy (Δ*G*^0^), and change in enthalpy (Δ*H*^0^) were calculated during optimization of the systems to assess the thermodynamic properties of the systems under study. Specifically, the changes in enthalpy and Gibbs free energy were evaluated *via*eqn (2)–(5).

2 Δ*H*_ads_ = *H*_complex_ − (*H*_gas_ + *H*_surface_)

3 Δ*G*_ads_ = *G*_complex_ − (*G*_gas_ + *G*_surface_)

where *H*_complex_ is the enthalpy of the adsorbed complex, *H*_gas_ is the enthalpy of the gas, *H*_surface_ is the enthalpy of the surface, where *G*_complex_ is the Gibbs free energy of the adsorbed complex, *G*_gas_ is the Gibbs free energy of the gas, and *G*_surface_ is the Gibbs free energy of the surface. The results are recorded in Table 6 for all the parameters, which include *ε*_0_ (electronic energy), *E*_tot_ (total energy), H_corr_ (thermal correction to enthalpy), *G*_corr_ (thermal correction to free energy), *ε*_0_ + *εZPE*, *ε*_*0*_ + *E*_tot_, *ε*_0_ + *H*_corr_, *ε*_0_ + *G*_corr_, Δ*G*^0^, and Δ*H*^0^. The total energy of all the systems was recorded to be approximately 0.2 hartrees for all the systems. The changes in the Gibbs free energy and enthalpy of all the systems were positive, portraying endothermic and nonspontaneous adsorption processes. This also indicates that the adsorption process is a physisorption type of adsorption that involves weak interactions between the cyanogenic gases and the Ag@Si_59_Y surface. The highest change in Gibbs free energy and enthalpy was observed for BrCN–Br–Ag@Si_59_Y, with values of 0.1267 hartree (79.5055 kcal mol^−1^) and 0.0023 hartree (1.4432 kcal mol^−1^), which characterize a more unfavorable or nonspontaneous adsorption process–despite the negative adsorption energy of −96.399 kcal mol^−1^ recorded for this system. This is the same for BrCN–N–Ag@Si_59_Y, with a value of 0.014 hartree (8.7851 kcal mol^−1^) and 0.0014 hartree (0.8785 kcal mol^−1^), which reveals a relatively unfavorable or nonspontaneous adsorption process, despite the negative adsorption energy of −83.867 kcal mol^−1^ recorded for this system. These two reaction conditions imply that the interaction between the Y and Br sites/Ag and N sites requires external energy, which is possibly a result of initial repulsive forces and weak bonding potential between the interacting species. Despite its nonspontaneity, its negative adsorption energy is related to the energy released, resulting in stable interactions and overcoming the initial energy barrier. However, bonding may involve weak forces such as van der Waals forces rather than strong covalent or electrostatic interactions. On the other hand, fewer positive values were observed for ClCN–Cl–Ag@Si_59_Y, ClCN–N–Ag@Si_59_Y, FCN–F–Ag@Si_59_Y and FCN–N–Ag@Si_59_Y, with corresponding positive adsorption energies. This shows that the interactions are energetically unfavorable and nonspontaneous, possibly as a result of repulsive forces and weak bonding. Thus, BrCN–Br–Ag@Si_59_Y and BrCN–N–Ag@Si_59_Y at the Br and N sites are the most thermodynamically favorable adsorption processes.

**Table 6:** Thermodynamic parameters for the adsorption of BrCN, ClCN, AND FCN on Ag@Si_59_Y *via* Br, Cl, F and N adsorption sites

Parameters	BrCN–Br–Ag@Si_59_Y	BrCN–N–Ag@Si_59_Y	ClCN–Cl–Ag@Si_59_Y	ClCN–N–Ag@Si_59_Y	FCN–F–Ag@Si_59_Y	FCN–N–Ag@Si_59_Y
*ε*_0_	−19924.88	−19924.86	−17811.01	−17811.01	−17450.67	−17450.67
*εZPE*	0.13	0.13	0.13	0.13	0.13	0.13
*E*_tot_	0.22	0.219	0.22	0.22	0.22	0.22
*H*_corr_	0.22	0.22	0.24	0.22	0.22	0.22
*G*_corr_	0.00	0.00	0.01	0.00	0.00	0.00
*ε*_0_ + *εZPE*	−19924.75	−19924.73	−17810.70	−17810.88	−17450.54	−17450.54
*ε*_0_ + *E*_tot_	−19924.66	−19924.64	−17810.45	−17810.79	−17450.45	−17450.45
*ε*_0_ + *H*_corr_	−19924.66	−19924.64	−17810.67	−17810.78	−17450.45	−17450.45
*ε*_0_ + *G*_corr_	−19924.88	−19924.85	−17812.22	−17811.01	−17450.67	−17450.67
Δ*G*^0^	0.127	0.01	0.02	0.014	0.01	0.01
Δ*H*^0^	0.00	0.00	0.00	0.00	0.00	0.00
*E*_ads_	−96.39	−83.87	89.63	89.93	95.31	95.31

### 3.9 Sensor mechanisms

The elucidation of the sensing mechanism includes a comprehensive analysis of the electronic structure and energy changes caused by the adsorption of cyanogenic gases on the functionalized nanocage. The key parameters include the work function (*ϕ*), charge transfer (*Q*_t_), fraction of electron transfer (Δ*N*), Δ*E* back-donation, dipole moment (*D*), and recovery time, which are carefully examined to evaluate the sensitivity, selectivity, and conductivity of the studied system.^28,33^ These parameters result from the optimization of the electron configuration of the system interaction, providing an understanding of the nanocage adsorption efficiency. This comprehensive analysis serves as a fundamental tool to explain the multiple mechanisms that detect the presence of materials and cyanogenic gases in particular.^10,13^ The ultimate goal is to develop sensors for the efficient and reliable detection of cyanogen gases with applications in environmental monitoring and public health.

#### 3.9.1 Work function (*ϕ*)

**Fig. 7:**
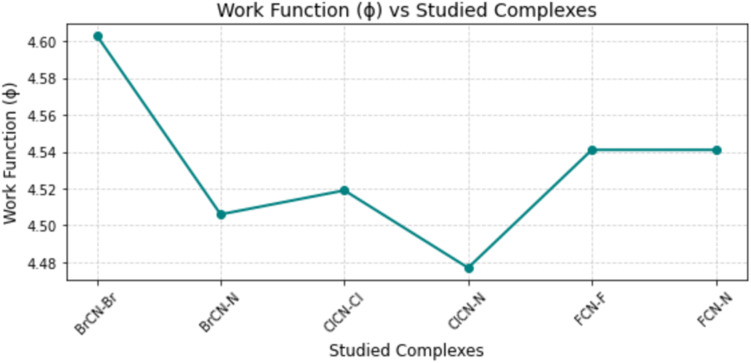
Comparison of work function values (in eV) for the studied systems, reflecting changes in electronic properties upon gas adsorption.

The work function represents the minimum energy required for electrons within a material to transfer from the conduction band to a free energy state.^9^ It plays a key role in describing a material's surface energy and electronic configuration, helping to evaluate its adsorption properties and revealing electron mobility within a given system.^9,27^ A higher work function can elucidate the suitability of a material as an insulator or semiconductor, whereas a lower value indicates the possible release of electrons due to thermal excitation upon pesticide adsorption.^35^ The calculated work function of this study is shown in Table 7 and Fig. 7, which shows a series of values for BrCN–Br–Ag@Si_59_Y, BrCN–N–Ag@Si_59_Y, and ClCN–Cl–Ag@Si_59_Y. ClCN–N–Ag@Si_59_Y, FCN–F–Ag@Si59Y, and FCN–N–Ag@Si_59_Y, which are 4.603, 4.506, 4.519, 4.477, 4.541, and 4.541 eV, respectively. This reveals that there is a high tendency of the systems to undergo conductivity, leading to the sensing of the studied gases. Even though the sensing of BrCN–Br–Ag@Si_59_Y behavior, as observed through this analysis, appears to be more profound, the differences observed between the systems in terms of their minimum energy required to undergo conduction are negligible. Hence, the conductivity potentials of the systems are closely related and show promising sensing behavior for the detection and adsorption of the studied gases.

**Table 7:** Sensor parameters for the studied complexes

Systems	*D*	*ϕ* (eV)	*Q*_t_	Δ*N*	Δ*E*	*τ* (s)
BrCN–Br–Ag@Si_59_Y	17.714	4.603	−0.701	2.421	0.506	8.64 × 10^−8^
BrCN–N–Ag@Si_59_Y	20.824	4.506	1.019	0.497	0.335	5.03 × 10^−8^
ClCN–Cl–Ag@Si_59_Y	20.034	4.519	−0.070	0.463	0.335	5.03 × 10^−8^
ClCN–N–Ag@Si_59_Y	23.368	4.477	1.017	4.955	0.503	4.87 × 10^−8^
FCN–F–Ag@Si_59_Y	18.978	4.541	1.784	3.461	0.503	4.65 × 10^−8^
FCN–N–Ag@Si_59_Y	18.999	4.541	1.072	3.461	0.503	4.65 × 10^−8^

#### 3.9.2 Charge transfer (*Q*_t_)

**Fig. 8:**
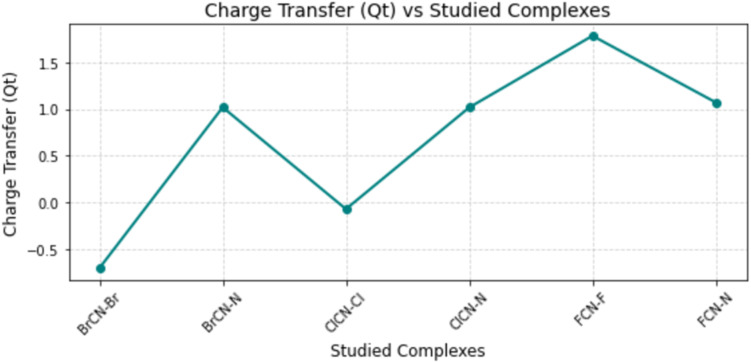
Net charge transfer (in electrons) between the adsorbed molecule and substrate, highlighting the direction and magnitude of electron movement.

In exploring sensor development for detecting these systems, the sensing mechanism is intricately tied to the observable interaction between the nanocages and the cyanogenic gases under investigation.^29^ This phenomenon is characterized by the acceptance or donation of electrons when a nanocomposite adsorbs gases, consequently instigating the transfer of charges between the involved molecules.^42^ The ensuing transfer of charges has profound implications for the electron conductivity within the system, arising from alterations in the structural properties of the molecules.^30^ To quantify this charge transfer, one can discern it by determining the difference in charge between the nanocage and the molecules of the respective gases. The resulting values, computed *via*eqn (3) and presented in Table 7, reveal notable distinctions in charge transfer among various systems. Notably, BrCN–Br–Ag@Si_59_Y, BrCN–N–Ag@Si_59_Y, and ClCN–Cl–Ag@Si_59_Y, ClCN–N–Ag@Si_59_Y, FCN–F–Ag@Si_59_Y, and FCN–N–Ag@Si_59_Y were observed with charge transfer values of −0.701, 1.019, −0.070, 1.017, 1.784, and 1.072*C*, respectively (see Fig. 8). The observed variations in charge transfer values at bromine and chlorine atoms compared with others likely stem from a combination of electronegativity, atomic size, chemical reactivity, adsorbate configuration, surface effects, and quantum mechanical considerations. For example, bromine and chlorine atoms are larger than some other atoms are, influencing the degree of orbital overlap with the adsorption sites.^43^ The complex interplay between the Ag@Si_59_Y surface and cyanogenic gases reveals a complex relationship wherein the charge transfer serves as a discernible parameter, shedding light on the electronic dynamics governing the sensor's functionality.

4 *Q*_*t*_ = *Q*_ads_ − *Q*_iso_

where *Q*_ads_ is the charge of the individual metal-doped surfaces and where *Q*_iso_ is the charge after interaction with cyanogenic gases.

#### 3.9.3 Fractions of electron transfer (Δ*N*) and back-donation (Δ*E*)

Fractional electron transfer is a subtle process in which electrons undergo partial transfer between nanocages when interacting with the molecules of the gases under investigation. The concept of giveback illustrates a related aspect of this intricate interaction.^32^ Specifically, reverse donation (Bak-donation) manifests itself as a unique form of electron transfer mechanism that occurs between the sensor system and the target analyte (in this case, the molecules of cyanogenic gases).^8,34^ This complex process occurs when the center of the sensor system donates an electron from its partially filled d orbital into the antibonding orbital associated with the gasses' molecule. To further understand these electron dynamics, both back donation and electron transfer require a certain energy threshold to cause changes in the electron density of the system.^9,13^ A noteworthy correlation was established, where higher hardness values correspond to reduced field effect transistor (FET) parameters and thus imply reduced electron transfer during adsorption.^35^ This observation highlights the importance of energy considerations in controlling electron transfer phenomena. Exploration of the electron density within molecules provides additional insights. A higher electron density within a molecular entity means an increased presence of closer electrons.^28^ Therefore, this increased electron density results in increased electrostatic repulsion between electrons, making adding or removing electrons from this region a more difficult task.

Table 7 summarizes the results of the interactions between the target analyte or molecule and the sensor system. This interaction ultimately leads to positron transfer, which involves the transfer of electrons from the sensor system to the analyte.^38^ As a result, an increase in the electron density of the analyte is observed, indicating that profound electronic transitions occur during this sensing process. To more fully understand and quantitatively evaluate the electronic changes inherent in the sensing process, eqn (5) and (6) provide a numerical specification. Such measurements have proven critical for interpreting sensor responses and detecting analytes, thereby enriching our understanding of the underlying electronic dynamics.^35^5
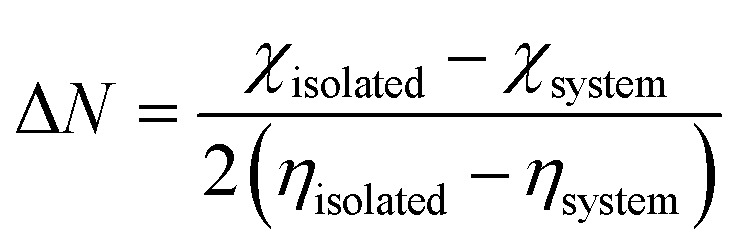
6
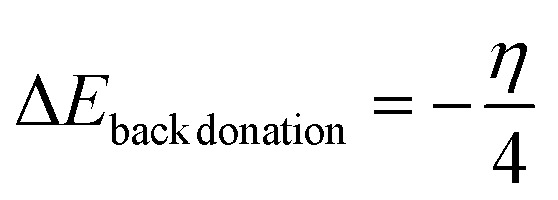


#### 3.9.4 Dipole moment

**Fig. 9:**
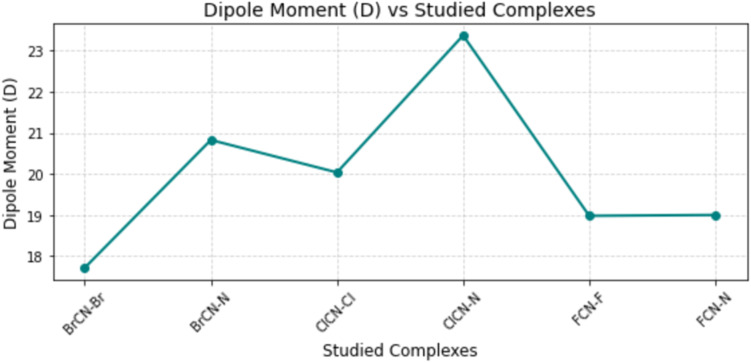
Variation of dipole moment (in Debye) for the studied complexes, indicating polarity differences influenced by the nature of halogen and adsorption site.

The dipole moment serves as a metric for measuring electrical charge separation, which is particularly important in sensor materials because of its influence on various factors, such as behavior, electrostatic interactions, electronic structure, and charge distribution.^12,31^ These aspects, in turn, play crucial roles in determining the performance and reactivity of sensor materials. Higher dipole moment values indicate a greater binding strength of the analytes. The results presented in Table 7 reveal that BrCN–Br–Ag@Si_59_Y, BrCN–N–Ag@Si_59_Y, and ClCN–Cl–Ag@Si_59_Y, ClCN–N–Ag@Si_59_Y, FCN–F–Ag@Si_59_Y, and FCN–N–Ag@Si_59_Y are characterized by electrical charge separations of 17.714, 20.824, 20.034, 23.368, 18.978, and 18.999 D, respectively (see Fig. 9). These results suggest that the electrical charge separation potential of all the investigated systems is high, as characterized by their high dipole moment values. The high dipole moments among the systems can be attributed to increased reactivity, adsorption, and sensitivity in their interactions with diazinon. The combined variations in work function (*ϕ*) and charge transfer (*Q*_t_) across the studied systems provide crucial insights into the selectivity and recovery behavior of the sensor. Changes in *ϕ* reflect alterations in surface electronic states upon gas adsorption. Gases that induce larger changes in *ϕ* cause more significant modifications in surface energy and electron mobility, enhancing the sensor's ability to distinguish between cyanogenic gases based on their electronic signatures.

Similarly, differences in *Q*_t_ represent the extent of electron exchange between the sensor and gas molecules, which is a key factor in determining the nature (physisorption or chemisorption) and strength of the interaction. Gases exhibiting higher *Q*_t_ values (*e.g.*, FCN–F–Ag@Si_59_Y) form stronger interactions with the sensing surface, leading to more pronounced signal responses and, consequently, higher selectivity. Moreover, the strength of these interactions has direct implications for the recovery time of the sensor. Stronger interactions—indicated by larger *Q*_t_ and higher Δ*ϕ*—typically require more energy for desorption, resulting in longer recovery times. In contrast, weaker interactions correlate with faster recovery, as the analyte detaches more easily from the sensor surface. Therefore, the interplay between *ϕ*, *Q*_t_, and recovery time not only explains the sensor's detection capability but also informs its practical applicability in real-time monitoring scenarios.

#### 3.9.5 Recovery time

The recovery time is characterized as the duration it takes for a sensor to revert to its initial baseline value following the abrupt removal of the measured variable. The recovery time, which is calculated *via*eqn (7), is centered on elucidating the sensor's responsiveness to analytes at room temperature.^27^7
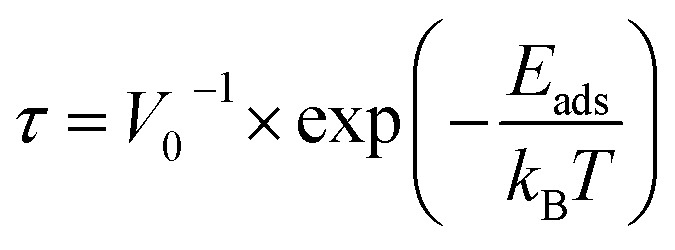
where; *τ* = recovery time, *V*_0_ = attempt frequency (10^12^ s^−1^), exp = exponential constant, *k*_B_ = Boltzmann constant, *E*_ads_ = absorption energy and *T* = absolute temperature.

**Fig. 10:**
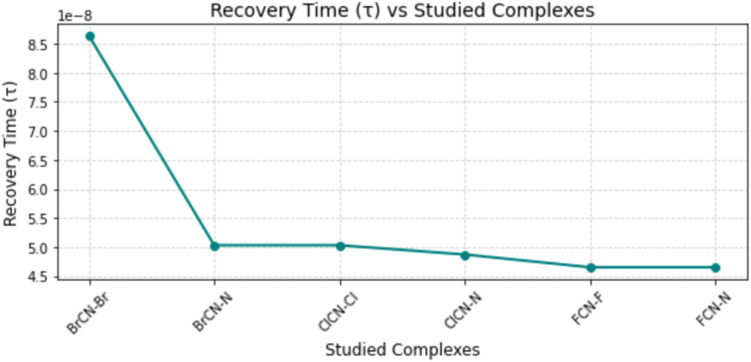
Estimated recovery time (in seconds) of the gas-sensor complexes, indicating the potential for reusability and sensing speed.

The correlation between recovery time and absorption energy (*E*_ads_) within each complex assumes paramount importance in the context of sensor applications, particularly in gas detection. A more negative *E*_ads_ value is indicative of a stronger interaction and heightened sensitivity to the analyte, as suggested by previous studies.^35^ Nonetheless, a discernible trade-off exists between sensitivity and recovery time. In essence, more negative *E*_ad_ values correspond to increased sensitivity but potentially prolonged recovery times owing to the robust interaction between the absorber and the attractant. Conversely, more positive *E*_ad_ values may result in shorter recovery times but potentially lower sensitivity.^43–48^ The recovery time at room temperature is detailed in Table 7. Notably, there is a direct relationship between the recovery time and the observed adsorption energy, indicating a strong chemical adsorption phenomenon between the nanocages and the gases. Specifically, the recovery times for systems exhibiting chemical adsorption are as follows: 8.64 × 10^−8^, 5.03 × 10^−8^, 5.03 × 10^−8^, 4.87 × 10^−8^, 4.65 × 10^−8^, and 4.65 × 10^−8^ for BrCN–Br–Ag@Si_59_Y, BrCN–N–Ag@Si_59_Y, ClCN–Cl–Ag@Si_59_Y, ClCN–N–Ag@Si_59_Y, FCN–F–Ag@Si_59_Y, and FCN–N–Ag@Si_59_Y, respectively. This explains why a more feasible (negative) adsorption energy necessitates a longer recovery time, thereby reinforcing the intricate relationship between the adsorption energy and desorption time within the studied systems (Fig. 10).

### 3.10 Electron localized function analysis (ELF)

**Fig. 11:**
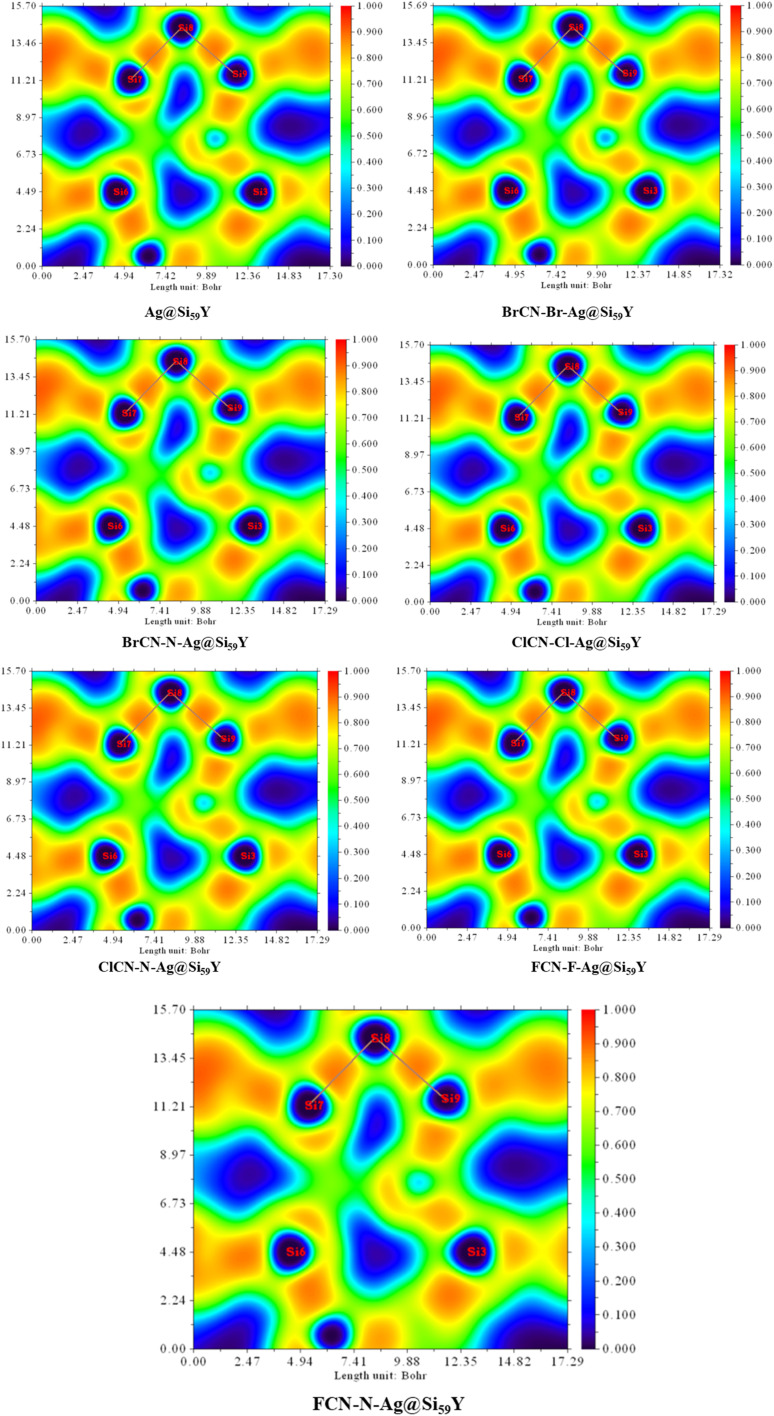
Electron Localization Function (ELF) plots of Ag@Si_59_Y and its gas-adsorbed complexes (BrCN–Br–Ag@Si_59_Y, BrCN–N–Ag@Si_59_Y, ClCN–Cl–Ag@Si_59_Y, ClCN–N–Ag@Si_59_Y, FCN–F–Ag@Si_59_Y, and FCN–N–Ag@Si_59_Y). The ELF scale ranges from 0 (blue, indicating delocalized electron density) to 1 (red, indicating high electron localization). Variations in localization between adsorbate and substrate indicate different interaction strengths, ranging from chemisorption to physisorption. The FCN–N–Ag@Si_59_Y plot shows weak interaction localized mainly around Si atoms, consistent with physisorption behavior.

The Electron Localization Function (ELF) plots for the various configurations—Ag@Si_59_Y, BrCN–Br–Ag@Si_59_Y, BrCN–N–Ag@Si_59_Y, ClCN–Cl–Ag@Si_59_Y, ClCN–N–Ag@Si_59_Y, FCN–F–Ag@Si_59_Y, and FCN–N–Ag@Si_59_Y as shown in Fig. 11—provide valuable insights into the electronic behavior and nature of bonding interactions between the adsorbed gas molecules and the Ag-decorated Si_59_Y nanocluster. ELF values range from 0 (blue, completely delocalized) to 1 (red, fully localized), helping us visualize regions of electron pairing such as covalent bonds, lone pairs, and localized cores. In the pristine Ag@Si_59_Y system, notable electron localization is evident around the silicon atoms, with a more diffuse distribution near the Ag site, suggesting potential for moderate adsorption interactions. Following adsorption, distinct changes in ELF topologies emerge. BrCN–Br and ClCN–Cl adsorptions show increased localization between the halogen and Ag atoms, reflecting stronger covalent or chemisorptive bonding. By contrast, the ELF around BrCN–N and ClCN–N interactions displays more diffuse regions at the interface, suggesting weaker, possibly physisorptive interactions—despite both configurations surprisingly sharing the same calculated adsorption energy of −17.571 kcal mol^−1^. The ELF differences help confirm that this numerical coincidence does not stem from a typographical error but from distinct orbital arrangements resulting in energetically similar but electronically dissimilar states. Further refining this understanding, the FCN–F–Ag@Si_59_Y system shows limited ELF overlap near the Ag–F interface, consistent with a weak van der Waals-type interaction due to fluorine's high electronegativity. Importantly, the FCN–N–Ag@Si_59_Y ELF map (newly shown) reinforces this trend; here, the nitrogen terminus of the adsorbed FCN molecule contributes to weak interaction with Ag, as seen from the sparse electron localization between Ag and the nearby Si atoms (Si_17_–Si_19_), and minimal overlap in the bonding region. This pattern supports physisorption behavior and underscores the critical role of adsorption orientation. Collectively, these ELF visuals provide a comprehensive qualitative validation of the adsorption energy calculations and reinforce the selectivity of Ag@Si_59_Y towards specific gas molecules and functional groups, with potential implications for reversibility in sensing applications.

## 4 Conclusions

This study underscores the vital role of computational modelling in addressing the hazardous nature of cyanogenic gases through density functional theory (DFT) calculations performed at the ωB97XD/GenECP/LanL2DZ/Def2SVP level of theory. Frontier molecular orbital (FMO) analysis revealed that the pristine Ag@Si_59_Y nanocluster possesses a high energy gap of 4.203 eV, indicative of a chemically stable and inert surface in its pristine state—yet one that exhibits significant reactivity upon gas adsorption. The marked reduction in energy gaps upon interaction with BrCN, ClCN, and FCN suggests enhanced surface sensitivity and electronic perturbation due to adsorption. Calculated adsorption energies revealed strong and selective affinities of the nanocluster for different gas configurations: −30.121 kcal mol^−1^ for BrCN–Br–Ag@Si_59_Y, −17.571 kcal mol^−1^ for BrCN–N–Ag@Si_59_Y and ClCN–Cl–Ag@Si_59_Y, −16.943 kcal mol^−1^ for ClCN–N–Ag@Si_59_Y, and −16.316 kcal mol^−1^ for both FCN–F–Ag@Si_59_Y and FCN–N–Ag@Si_59_Y. These values point to favorable adsorption energies, especially for BrCN derivatives. Natural Bond Orbital (NBO) analysis further confirmed strong donor–acceptor interactions—particularly π → LP* transitions—highlighting the importance of charge delocalization and stabilization within the host–guest systems. Electron Localization Function (ELF) analysis was employed to visually probe the nature of electron distribution and bonding. The ELF maps exhibited moderate to high electron localization around the Ag–Si and Ag–gas interaction regions in strongly adsorbed systems, such as BrCN–Br–Ag@Si_59_Y and FCN–N–Ag@Si_59_Y. These patterns suggest partial covalent characteristics superimposed on predominantly noncovalent interactions, complementing insights from QTAIM and validating the classification of interaction regimes.

Noncovalent interaction (NCI) plots further confirmed the role of hydrogen bonding and electrostatic forces in stabilizing the adsorption complexes. BrCN displayed the strongest overall interaction profile, consistent with its highest adsorption energy and extended recovery time—indicative of chemisorption. Moreover, the high dipole moments observed across all complexes underscore significant charge polarization, enhancing their prospects for real-time gas sensing applications. Collectively, this comprehensive investigation, integrating energy-based descriptors, real-space functionals, and electronic structure tools, elucidates the adsorption behavior of toxic cyanogenic gases on Ag@Si_59_Y. The findings contribute valuable design principles for developing advanced nanostructured materials for selective gas sensing and environmental monitoring.

## Authorship contribution

I. J. M.: result extraction, conceptualization, writing and manuscript first draft, resources, review, and editing. G. E. M: design, validation, supervision, and editing validation, investigation, analysis, writing, and manuscript final draft. E. O. U.: writing and manuscript first draft, resources. Z. A. A. A.: result extraction, writing and manuscript first draft. T. A. A. M. A.: result extraction, writing and manuscript final draft.

## Conflicts of interest

All authors declare zero financial or inter-personal conflict of interest that could have influenced the research work or results reported herein.

## Supplementary Material

RA-015-D5RA03374H-s001

## Data Availability

All data generated or analyzed during this study are included in this article and its ESI file.†

## References

[cit1] Shifrin N. S., Beck B. D., Gauthier T. D., Chapnick S. D., Goodman G. (1996). Chemistry, toxicology, and human health risk of cyanide compounds in soils at former manufactured gas plant sites. Regul. Toxicol. Pharmacol..

[cit2] Yang X., Lan L., Zhao Z., Zhou S., Kang K., Song H. (2022). *et al.*, A review on cyanide gas elimination methods and materials. Molecules.

[cit3] Zengel D., Koch P., Torkashvand B., Grunwaldt J. D., Casapu M., Deutschmann O. (2020). Emission of toxic HCN during NOx removal by ammonia SCR in the exhaust of lean-burn natural gas engines. Angew. Chem., Int. Ed..

[cit4] Yuan S., Zhou Z. J., Li J., Chen X. L., Wang F. C. (2010). HCN and NH3 released from biomass and soybean cake under rapid pyrolysis. Energy Fuels.

[cit5] Ma J., Dasgupta P. K. (2010). Recent developments in cyanide detection: a review. Anal. Chim. Acta.

[cit6] Scholes C. A. (2020). Hydrogen cyanide recovery by membrane gas separation. Chem. Eng. J..

[cit7] Unimuke T. O., Mbonu I. J., Louis H., Mathias G. E., Hossain I., Ikenyirimba O. J. (2024). *et al.*, Surface engineering of non-platinum-based electrocatalysts for sustainable hydrogen production: encapsulation, doping, and decoration approach. Int. J. Hydrogen Energy.

[cit8] Mohammadi M. D., Abdullah H. Y., Louis H., Etim E. E., Edet H. O. (2023). Evaluating the detection potential of C59X fullerenes (X = C, Si, Ge, B, Al, Ga, N, P, and As) for H2SiCl2 molecule. J. Mol. Liq..

[cit9] Chen Y., Han J., Yang X. (2023). DFT simulation of structure stability and nitrogen oxide adsorption for nitrogen and oxygen co-modified carbon nanotubes. Surf. Interfaces.

[cit10] Karimi N., Sardroodi J. J., Rastkar A. E. (2022). The adsorption of NO2, SO2, and O3 molecules on the Al-doped stanene nanotube: a DFT study. J. Mol. Model..

[cit11] Jasim S. A., Ahmed E., Al-Ghazaly S. M., Al-Seady M. A., Abduljalil H. M. (2022). Study adsorption ability of pure single walled carbon nano tube to detection some toxic gases using DFT calculation. AIP Conf. Proc..

[cit12] Ogunwale G. J., Louis H., Unimuke T. O., Mathias G. E., Owen A. E., Edet H. O. (2023). *et al.*, Interaction of 5-fluorouracil on the surfaces of pristine and functionalized Ca12O12 nanocages: an intuition from DFT. ACS Omega.

[cit13] Solimannejad M., Anjiraki A. K., Kamalinahad S. (2017). Sensing performance of Cu-decorated Si_12_C_12_ nanocage towards toxic cyanogen gas: a DFT study. Mater. Res. Express.

[cit14] Abraham B. M. (2021). Adsorption of cyanogen halides (X-CN; X = F, Cl and Br) on pristine and Fe, Mn doped C60: a highly potential gas sensor. Mater. Today Commun..

[cit15] Castrucci P., Scarselli M., De Crescenzi M., Diociaiuti M., Chaudhari P. S., Balasubramanian C. (2006). *et al.*, Silicon nanotubes: synthesis and characterization. Thin Solid Films.

[cit16] Ghosh R. S. , Dzombak D. A. and Wong-Chong G. M., Physical and chemical forms of cyanide, in. Cyanide in Water and Soil, CRC Press, Boca Raton, 2005, pp. 27–36

[cit17] Wang L. M., Pal R., Huang W., Zeng X. C., Wang L. S. (2009). Tuning the electronic properties of the golden buckyball by endohedral doping: M@Au_16_− (M = Ag, Zn, In). J. Chem. Phys..

[cit18] Wang L. M., Bulusu S., Huang W., Pal R., Wang L. S., Zeng X. C. (2007). Doping the golden cage Au_16_− with Si, Ge, and Sn. J. Am. Chem. Soc..

[cit19] Wang L. M., Bai J., Lechtken A., Huang W., Schooss D., Kappes M. M. (2009). *et al.*, Magnetic doping of the golden cage cluster M@Au_16_− (M = Fe, Co, Ni). Phys. Rev. B:Condens. Matter Mater. Phys..

[cit20] Manzoor D., Krishnamurty S., Pal S. (2016). Endohedrally doped gold nanocages: efficient catalysts for O2 activation and CO oxidation. Phys. Chem. Chem. Phys..

[cit21] Manzoor D., Krishnamurty S., Pal S. (2016). Contriving a catalytically active structure from an inert conformation: a density functional investigation of Al, Hf, and Ge doping of Au20 tetrahedral clusters. J. Phys. Chem. C.

[cit22] Manzoor D., Pal S. (2015). Reactivity and catalytic activity of hydrogen atom chemisorbed silver clusters. J. Phys. Chem. A.

[cit23] Wang L. M., Bulusu S., Zhai H. J., Zeng X. C., Wang L. S. (2007). Doping golden buckyballs: Cu@Au_16_− and Cu@Au_17_− cluster anions. Angew. Chem., Int. Ed..

[cit24] Frisch M. J. , Trucks G. W., Schlegel H. B., Scuseria G. E., Robb M. A. and Cheeseman J. R., et al., Gaussian 16 Rev. C.01, Gaussian Inc., Wallingford, CT, 2016

[cit25] Romano E., Manzur M. E., Iramain M. A., Brandán S. A. (2023). Effect of long-range corrections on intermolecular interactions and vibrational assignments of ethylene oxide dimer: a combined DFT and SQFF study. Eur. J. Theor. Appl. Sci..

[cit26] Glendening E. D., Landis C. R., Weinhold F. (2019). NBO 7.0: new vistas in localized and delocalized chemical bonding theory. J. Comput. Chem..

[cit27] Zhurko G. A. , Zhurko D. A.. Chemcraft—Graphical program for visualization of quantum chemistry computations. Chemcraft Acad, Version. 2005;1

[cit28] Lu T., Chen F. (2012). Multiwfn: a multifunctional wavefunction analyzer. J. Comput. Chem..

[cit29] Edwards P. M. (2002). Origin 7.0: scientific graphing and data analysis software. J. Chem. Inf. Comput. Sci..

[cit30] Humphrey W., Dalke A., Schulten K. (1996). VMD: visual molecular dynamics. J. Mol. Graphics.

[cit31] Anis I., Majeed S., Bilkees U., Mir J. M., Malik S. A., Dar M. A. (2025). Impact of hetero-atom doping on electronic structure and reactivity of anionic Al_13_− cluster: a combined density functional theory and global optimization investigation. Struct. Chem..

[cit32] Agwamba E. C., Mathias G. E., Louis H., Ikenyirimba O. J., Unimuke T. O., Ahuekwe E. F. (2023). *et al.*, Single metal-doped silicon (Si59X; X = Nb, Mo, Y, Zr) nanostructured as nanosensors for N-nitrosodimethylamine (NDMA) pollutant: intuition from computational study. Mater. Today Commun..

[cit33] Mohammadi M. D., Abdullah H. Y., Biskos G., Bhowmick S. (2023). Adsorbing CNCl on pristine, C-, and Al-doped boron nitride nanotubes: a density functional theory study. Comput. Theor. Chem..

[cit34] Silva A. L. P., Varela Júnior J. D. J. G. (2023). MB11N12 (M = Fe–Zn) nanocages for cyanogen chloride detection: a DFT study. J. Inorg. Organomet. Polym. Mater..

[cit35] Dai J., Yuan J., Giannozzi P. (2009). Gas adsorption on graphene doped with B, N, Al, and S: a theoretical study. Appl. Phys. Lett..

[cit36] Ekereke E. E., Ikechukwu O. C., Louis H., Gber T. E., Charlie D. E., Ikeuba A. I. (2023). *et al.*, Quantum capacitances of alkaline earth metals: Be, Ca, and Mg integrated on Al12N12 and Al12P12
nanostructured—insight from DFT approach. Monatsh. Chem..

[cit37] Nwobodo I. C., Louis H., Unimuke T. O., Ikenyirimba O. J., Iloanya A. C., Mathias G. E. (2023). *et al.*, Molecular simulation of the interaction of diclofenac with halogen (F, Cl, Br)-encapsulated Ga12As12 nanoclusters. ACS Omega.

[cit38] Hussain S., Shahid Chatha S. A., Hussain A. I., Hussain R., Mehboob M. Y., Gulzar T. (2020). *et al.*, Designing novel Zn-decorated inorganic B12P12 nanoclusters with promising electronic properties: a step forward toward efficient CO2 sensing materials. ACS Omega.

[cit39] Mohammadi M. D., Louis H., Ikenyirimba O. J., Mathias G. E., Unimuke T. O., Rasaki M. E. (2023). Endohedral doping of Ca12O12-X (X = Zn, Cd, and Hg) as hydrogen storage materials. Int. J. Hydrogen Energy.

[cit40] Unimuke T. O., Louis H., Ikenyirimba O. J., Mathias G. E., Adeyinka A. S., Nasr C. B. (2023). High throughput computations of the effective removal of liquified gases by novel perchlorate hybrid material. Sci. Rep..

[cit41] Barpanda P., Fanchini G., Amatucci G. G. (2011). Structure, surface morphology and electrochemical properties of brominated activated carbons. Carbon.

[cit42] Lin Z. D., Young S. J., Hsiao C. H., Chang S. J. (2013). Adsorption sensitivity of Ag-decorated carbon nanotubes toward gas-phase compounds. Sens. Actuators, B.

[cit43] Yang X., Li L., Yan F. (2010). Polypyrrole/silver composite nanotubes for gas sensors. Sens. Actuators, B.

[cit44] Fozia S., Hassan A., Reshi S. A., Singh P., Bhat G. A., Dixit M. (2023). *et al.*, Boosting CO2 activation and reduction by engineering the electronic structure of graphitic carbon nitride through transition metal-free single-atom functionalization. J. Phys. Chem. C.

[cit45] Anis I., Amin S., Rather G. M., Dar M. A. (2023). N2 activation and reduction on graphdiyne supported single, double, and triple boron atom catalysts: a first principles investigation. ChemistrySelect.

[cit46] Dar M. A. (2023). Implications of the pore size of graphitic carbon nitride monolayers on the selectivity of dual-boron atom catalysts for the reduction of N2 to urea and ammonia: a computational investigation. Inorg. Chem..

[cit47] Rasool A., Anis I., Bhat S. A., Dar M. A. (2023). Optimizing the NRR activity of single and double boron atom catalysts using a suitable support: a first principles investigation. Phys. Chem. Chem. Phys..

[cit48] Hassan A., Dar M. A. (2023). Understanding the activity of single atom catalysts for CO2 reduction to C2 products: a high throughput computational screening. New J. Chem..

