# Supplementary material for: Chemical effect of silver (Ag) and yttrium (Y) co-doping on silicon-based fullerene (Ag@Si59Y) sensor nanostructures: a computational adsorption study of cyanogenic halide gases
Source: RSC Adv. 2025 Jul 28;15(33):26693–709. doi: 10.1039/d5ra03374h (PMC12301876; doi:10.1039/d5ra03374h)
Supplement: RA-015-D5RA03374H-s001 [file RA-015-D5RA03374H-s001.pdf]

Supplementary Information (SI) for RSC Advances.
This journal is © The Royal Society of Chemistry 2025

Chemical effect of Silver (Ag) and Yttrium (Y) co-doping of Silicon-based fullerene (Ag@Si_{59}Y) sensor nanostructured: A computational adsorption study of cyanogenic halide gases

Idongesit J. Mbonu [1], Gideon E. Mathias [2,5*], Emily O. Udowa [1], Zainab Abbas Abd Alhassan [3], and Thamer A.A.M. Alalwani [4]

[1] Department of Chemistry, Federal University of Petroleum Resources, Effurun, Nigeria

[2] Department of Pure and Chemistry, University of Calabar, Calabar, Nigeria

[3] Mazaya University College, Dhiqar Iraq

[4] Radiological Techniques Department, College of Health and Medical Techniques, Al-Mustaqbal University, 51001, Babylon, Iraq

[5] Biomedical and Computational Chemistry Group, University of Calabar, Calabar Nigeria

[*]Corresponding author's email:
mathiasgideon610@gmail.com

Supporting Information

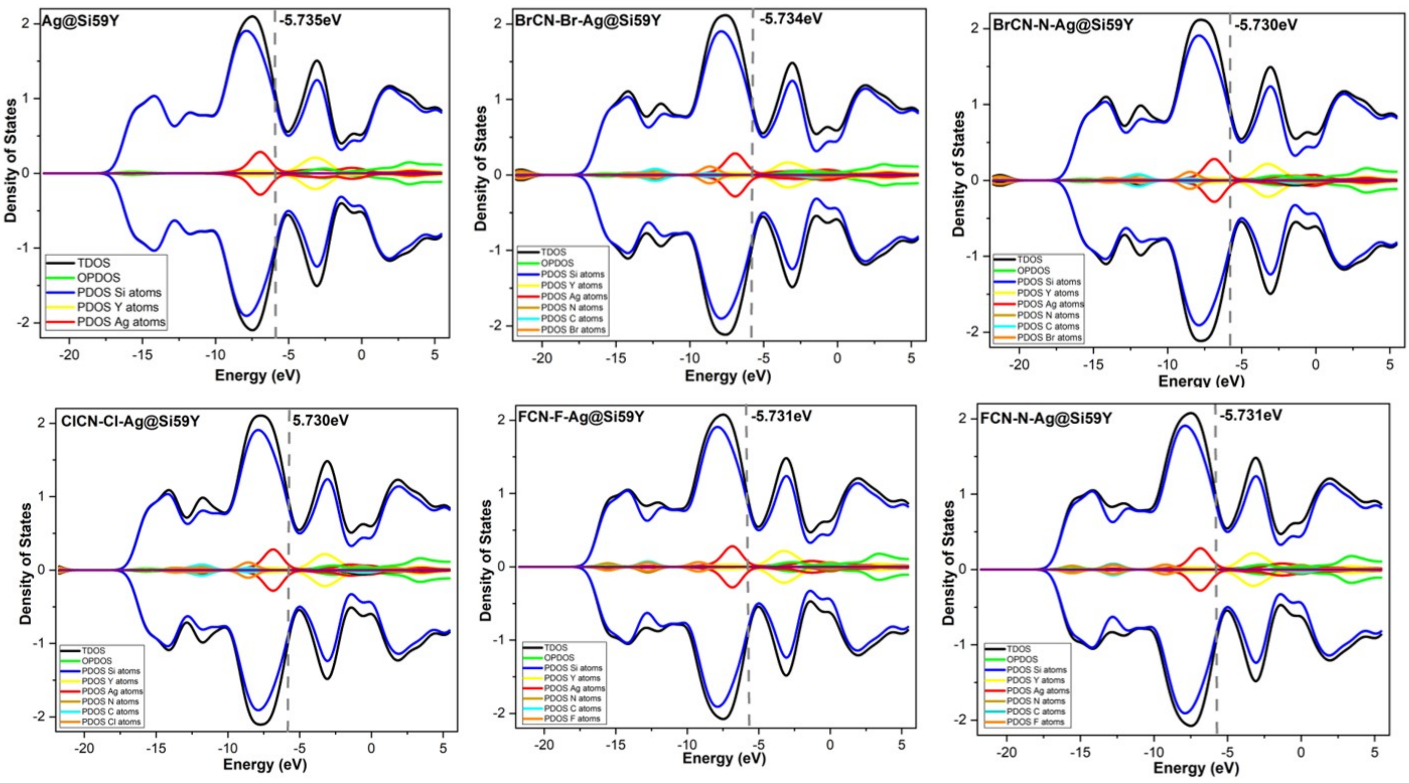

Figure S1. DOS plot for the system and complexes
